# Association Between Sulfur Dioxide and Daily Inpatient Visits With Respiratory Diseases in Ganzhou, China: A Time Series Study Based on Hospital Data

**DOI:** 10.3389/fpubh.2022.854922

**Published:** 2022-03-31

**Authors:** Xingye Zhou, Yanfang Gao, Dongming Wang, Weihong Chen, Xiaokang Zhang

**Affiliations:** ^1^School of Public Health and Health Management, Gannan Medical University, Ganzhou, China; ^2^Department of Occupational and Environmental Health, School of Public Health, Tongji Medical College, Huazhong University of Science and Technology, Wuhan, China; ^3^Key Laboratory of Environment and Health, Ministry of Education, Ministry of Environmental Protection, State Key Laboratory of Environmental Health (Incubating), School of Public Health, Tongji Medical College, Huazhong University of Science and Technology, Wuhan, China

**Keywords:** sulfur dioxide, air pollution, respiratory disease, inpatient visits, time-series analysis, generalized additive models, distributed lag non-linear models

## Abstract

**Background:**

Sulfur dioxide (SO_2_) has been reported to be related to the mortality of respiratory diseases, but the relationship between SO_2_ and hospital inpatient visits with respiratory diseases and the potential impact of different seasons on this relationship is still unclear.

**Methods:**

The daily average concentrations of air pollutants, including SO_2_ and meteorological data in Ganzhou, China, from 2017 to 2019 were collected. The data on daily hospitalization for respiratory diseases from the biggest hospital in the city were extracted. The generalized additive models (GAM) and the distributed lag non-linear model (DLNM) were employed to evaluate the association between ambient SO_2_ and daily inpatient visits for respiratory diseases. Stratified analyses by gender, age, and season were performed to find their potential effects on this association.

**Results:**

There is a positive exposure-response relationship between SO_2_ concentration and relative risk of respiratory inpatient visits. Every 10 μg/m^3^ increase in SO_2_ was related to a 3.2% (95% CI: 0.6–6.7%) exaltation in daily respiratory inpatient visits at lag3. In addition, SO_2_ had a stronger association with respiratory inpatient visits in women, older adults (≥65 years), and warmer season (May-Oct) subgroups. The relationship between SO_2_ and inpatient visits for respiratory diseases was robust after adjusting for other air pollutants, including PM_10_, NO_2_, O_3_, and CO.

**Conclusion:**

This time-series study showed that there is a positive association between short-term SO_2_ exposure and daily respiratory inpatient visits. These results are important for local administrators to formulate environmental public health policies.

## Introduction

Air pollution is one of the most important public health problems around the world (1). Sulfur dioxide (SO_2_), mainly comes from various industrial processes, transportation, and vehicles, power plants, and fuel (coal) combustion, is one of the most common and irritant air pollutants in developing countries and industrialized areas (2–5). Several epidemiological studies have revealed that SO_2_ exposure is related to human respiratory health (6, 7), including stimulating the respiratory tract (8), leading to the decline of pulmonary function (9, 10), and the increased mortality due to respiratory diseases (11, 12). However, there are few studies on the relationship between SO_2_ and respiratory morbidity in developing countries. Inpatient visit is an important indicator of morbidity and has been widely used to assess the adverse effects associated with atmospheric pollutants (13). Therefore, it will be helpful to understand the impact of SO_2_ on the respiratory system by evaluating the relationship between ambient air SO_2_ and the number of hospitalized cases of the respiratory system.

Ganzhou is located in the southern part of Jiangxi Province. The ambient temperature and lifestyle of Ganzhou are typical of southern China. In 2019, the mean concentrations of PM_10_, SO_2_, NO_2_, O_3_, and CO in Ganzhou were 51, 12, 22, 74, and 1.2 mg/m^3^, respectively. Except for SO_2_, the concentrations of other air pollutants in Ganzhou were lower than the mean concentration of 337 Chinese cities (63 μg/m^3^ for PM_10_, 11 μg/m^3^ for SO_2_, 27 μg/m^3^ for NO_2_, 148 μg/m^3^ for O_3_, and 1.4 mg/m^3^ for CO, respectively) (14). Compared with lower levels of other air pollutants, the concentration of SO_2_ in Ganzhou is higher than that of the national average in China, drawing more attention to the potential respiratory health damage caused by SO_2_.

In this study, the daily average concentrations of air pollutants, including SO_2_ from 2017 to 2019, were collected from the local Environmental Protection Bureau. The respiratory inpatient visits were recorded during the same period in the biggest hospital in Ganzhou. We analyzed the association between the level of SO2 exposure and the daily respiratory inpatient visit, investigated populations more sensitive to SO_2_ exposure, assessed the potential impact of seasonal changes on SO_2_ lagging effects, and explored the exposure-response relationship between SO_2_ concentrations and different population subgroups.

## Methods

### Study Area

Ganzhou (24°29′- 27°09′ N; 113°54′- 116°38′ E) is located in the southern region of China. The terrain of the area is dominated by mountains and hills. Ganzhou has a total population of 9.82 million and an area of 39,379 km^2^, accounting for 23.6% of the total area of Jiangxi. The area is characterized by a subtropical monsoon climate. The average annual rainfall in Ganzhou in 2020 is 1,706.4 mm; the average temperature is 19.9°C; the average sunshine hours is 1,637.9 h (15).

### Data Collection

In this time-series analysis study, we extracted the daily respiratory inpatient visits from January 1, 2017, to December 31, 2019, from the medical database of the largest hospital (the First Affiliated Hospital of Gannan Medical University) in Ganzhou. A total of 9,668 respiratory inpatient visits were recorded during the study period. The respiratory inpatients were identified by the primary code of admission diagnosis (ICD-10: J00–J99). The concentration data of atmospheric pollutants in this study came from the Ganzhou Environmental Protection Bureau. There were five air monitoring stations in the city, and the mean daily concentrations of ambient PM_10_, SO_2_, NO_2_, O_3_, and CO were collected by these fixed monitoring stations. The daily temperature and humidity data during the study period were collected from the Ganzhou Meteorological Bureau. The daily air pollutant concentrations and meteorological data were not missing.

### Statistical Analysis

Daily inpatient visits are generally considered to be rare events and have a Poisson distribution. Therefore, this study used the generalized additive model (GAM) and the distributed lag non-linear model (DLNM) to investigate the relationship between SO_2_ and respiratory daily hospitalizations. Due to potential non-linear effects, natural spline functions were used to control the confounding factors, such as long-term trends, relative humidity, temperature, day of the week effect, and the effect of holidays. The GAM was selected to evaluate the health effects of SO_2_ under different lag days (including single-day lag effects and multi-day lag effects). Referring to previous studies (16, 17), the model of the hysteresis effect of SO_2_ is as follows:


$$\begin{array}{l} Log\left[E\left(Y_{t}\right)\right]=\beta X_{t}+ns\left(time,\;df=7/per\;year\right)+ns\left(Temp,df=3\right)\\ \;+ns\left(RH,df=3\right)+DOW+Holiday+\alpha \end{array}$$

where E(Y_t_) means the expected number of inpatient visits for the respiratory system at day t; X_t_ and β represent the concentration of SO_2_ in the atmosphere on day t and the regression coefficient, respectively; ns is natural spline function; df refers to the degrees of freedom; DOW means the day of the week; Holiday indicates the effect of holidays; α refers to a constant.

The DLNM was used to reflect the exposure-response relationship between SO_2_ concentrations and the relative risk of respiratory inpatient visits based on the hysteresis effect. The cross-basis function can combine the two dimensions of atmospheric pollutant concentration and lag days. Referring to related research (18–20), the model of the exposure-response relationship is as follows:


$$\begin{array}{l} Log\left(u_{t}\right)=\beta X_{t,i}+ns\left(time,df=7/peryear\right)+ns\left(Temp,df=3\right)\\ \;+ns\left(RH,df=3\right)+DOW+Holiday+\alpha \end{array}$$

where E(Y_t_) denotes the expected number of respiratory inpatient visits at lag day t; X_t, I_ and β represent the cross-basis function of SO_2_ and the regression coefficient, respectively.

Several analyses were adopted to investigate the relation between SO_2_ and respiratory inpatient visits. Firstly, single-pollutant model, including single-day lag (from lag0, which meant current day estimated effect, to lag7, which meant the previous 7th day estimated effect) and multi-day lag (from lag0–1, which represented the mean of the current day effect and lag1 effect, to lag0–7, which represented the mean of the current day effect and the previous 7 days' effects), was selected to research the lag pattern of SO_2_. Secondly, the expose-response relationship between the SO_2_ concentration and the relative risk of respiratory inpatient visits was plotted. Thirdly, the stratified analysis was selected to explore the relationship between the SO_2_ level and the inpatient visits for respiratory diseases in different gender, ages, and season subgroups. Finally, the multi-pollutant model was used to evaluate the stability of the single pollutant model after adjusting for other atmospheric pollutants, such as PM_10_, NO_2_, O_3_, and CO.

The results of the exposure-response relationship were presented as the relative risk (RR) of respiratory inpatient visits caused by SO_2_ exposure as a continuous variable. The rest of the results were presented as the percentage changes (PC) and 95% CI in daily respiratory inpatient visits each 10 μg/m^3^ increment of SO_2_ levels. In this study, two-sided *p* < 0.05 was considered statistically significant. All statistical analyses were conducted in R 4.0.2 using the “mgcv” and “dlnm” packages.

## Results

Table 1 presents descriptive results for ambient air pollutant concentrations, meteorological parameters, and daily respiratory inpatient visits. During the 1,095 days from January 1, 2017, to December 31, 2019, the average daily concentrations (standard deviation) of atmospheric pollutants were 62.7 (34.6) μg/m^3^ for PM_10_, 18.5 (11) μg/m^3^ for SO_2_, 24.2 (13) μg/m^3^ for NO_2_, 71.7 (34.5) μg/m^3^ for O_3_, and 1.3 (0.3) mg/m^3^ for CO, respectively. Moreover, the daily mean relative humidity and temperature were 74% and 19.6°C, respectively. A total of 9,668 respiratory inpatient visits were recorded from 2017 to 2019; the daily mean count of inpatient visits was 9. Women accounted for ∽34.1% of all the cases, and younger people (<65 years) accounted for ∽48.1% of all the cases.

**Table 1:** Data for ambient air pollutants, weather conditions, and respiratory inpatient visits in Ganzhou from 2017 to 2019.

		**Mean ±SD**	**Minimum**	**P (25)**	**Median**	**P (75)**	**Maximum**
PM_10_ (μg/m^3^)		62.7 ± 34.6	11	38	54	79	258
SO_2_ (μg/m^3^)		18.5 ± 11.0	3	11	16	23	73
NO_2_ (μg/m^3^)		24.2 ± 13.0	8	15	20	29	84
O_3_ (μg/m^3^)		71.7 ± 34.5	5	46	70	94	194
CO (mg/m^3^)		1.3 ± 0.3	0.6	1.1	1.3	1.5	2.9
Temperature (°C)		19.6 ± 8.1	0	13	21	27	32
Relative humidity (%)		74.0 ± 12.5	36	64	74	84	99
Daily inpatients visits		8.8 ± 3.9	0	6	9	11	25
Gender	Male	5.8 ± 2.9	0	4	6	8	18
	Female	3.0 ± 1.9	0	2	3	4	10
Age	≥65	4.6 ± 2.6	0	3	4	6	18
	<65	4.2 ± 2.4	0	3	4	6	15
Season	Cold	9.1 ± 4.1	0	6	9	12	25
	Warm	8.5 ± 3.6	1	6	8	11	21

The correlations between meteorological parameters and atmospheric pollutants are shown in Table 2. All the correlation coefficients between the meteorological factors and the atmospheric pollutants were statistically significant. The daily concentration of SO_2_ was positively correlated with other air pollutants (PM_10_, NO_2_, O_3_, and CO) and temperature (correlation coefficient = 0.07–0.71) and negatively correlated with relative humidity (correlation coefficient = −0.41). Moreover, PM_10_ and NO_2_ had positive associations with other air pollutants (correlation coefficient = 0.12–0.71), while there was a negative correlation between CO and O_3_ (correlation coefficient = −0.16). The temperature was also negatively associated with relative humidity (correlation coefficient = −0.31).

**Table 2:** Spearman's correlation of air pollutants and weather conditions.

	**PM_**10**_**	**SO_**2**_**	**NO_**2**_**	**O_**3**_**	**CO**	**Temperature**	**Relative humidity**
PM_10_	1	0.71	0.69	0.38	0.29	−0.13	−0.46
SO_2_		1	0.56	0.30	0.12	0.07	−0.41
NO_2_			1	−0.07	0.38	−0.44	−0.13
O_3_				1	−0.16	0.40	−0.65
CO					1	−0.39	0.18
Temperature						1	−0.31
Relative humidity							1

*All correlation coefficients are statistically significant (p <0.05)*.

Table 3 shows the percent changes in daily respiratory inpatient visits associated with a 10 μg/m^3^ increase in SO_2_ on different lag days in different gender and age subgroups. The delayed effects of SO_2_ were significant at lag3, lag4, lag0–3, lag0–4, lag0–5, lag0–6, and lag0–7, with the maximum effect observed at lag4 (PC: 3.4%; 95% CI: 0.4–6.4%) in single-lag days. The maximum effect of multi-lag days was at lag 0:5 with 6.9% (95% CI: 1.6–12.5%). For men, the association between SO_2_ exposure and respiratory admission was not statistically significant at any lag days. The SO_2_ concentration was significantly correlated with women at lag1, lag4, and all the multi-lag days, with the largest effect observed at lag1 (PC: 7.1%; 95% CI: 1.9–12.6%) in the single-lag day model. For younger people (<65 years), there is no statistically significant relationship between SO_2_ and inpatient visits for respiratory diseases at any lag days. For the elderly (≥65 years), daily respiratory inpatient visits were significantly associated with SO_2_ concentration at lag1, lag4, and all the multi-lag days except lag0:7; the strongest association was observed at lag1 (PC: 5.5%; 95% CI: 1–10.2%) in single-lag day model.

**Table 3:** Percentage change (95% CI) of inpatient visits for respiratory diseases per 10 μg/m^3^ increase in concentrations of SO_2_ for different lag days in the single-pollutant model.

**Lag type**	**Lag day**	**Total**	**Male**	**Female**	** <65**	**≥65**
Single-lag days	0	1.8 (−1.5/5.3)	0.7 (−3.3/4.9)	3.7 (−1.9/9.5)	−0.7 (−5.0/3.7)	4.7 (−0.2/9.8)
	1	2.9 (−0.2/6.1)	0.9 (−2.7/4.8)	**7.1 (1.9/12.6)**	0.9 (−3.2/5.1)	**5.5 (1.0/10.2)**
	2	2.1 (−0.8/5.2)	1.4 (−2.2/5.1)	2.7 (−2.2/7.9)	1.5 (−2.5/5.5)	2.3 (−1.0/5.7)
	3	**3.2 (0.6/6.7)**	2.7 (−0.9/6.4)	4.5 (−0.4/9.5)	3.8 (−0.1/7.9)	2.7 (−0.6/6.1)
	4	**3.4 (0.4/6.4)**	2.1 (−1.5/5.8)	**5.1 (0.2/10.2)**	0.8 (−3.1/4.8)	**4.5 (1.2/7.9)**
	5	0.9 (−2.0/3.9)	−0.7 (−4.2/2.9)	3.8 (−1.1/8.9)	−0.3 (−4.2/3.7)	1.6 (−1.6/4.9)
	6	−1.4 (−4.2/1.6)	−1.5 (−5.0/2.1)	−1.4 (−6.1/3.5)	0.6 (−3.3/4.6)	−3.2 (−6.3/−0.1)
	7	0.6 (−2.3/3.6)	1.4 (−2.2/5.0)	−1.31 (−5.9/3.5)	2.0 (−1.8/6.1)	−0.9 (−4.1/2.3)
Multi-lag days	0–1	3.4 (−0.4/7.2)	1.1 (−2.4/5.7)	**7.7 (1.3/14.4)**	0.2 (−4.7/5.3)	**7.1 (1.5/13.0)**
	0–2	3.5 (−0.3/8.3)	1.8 (−3.2/7.0)	**7.9 (0.9/15.5)**	1.1 (−4.3/6.7)	**7.2 (1.1/13.7)**
	0–3	**5.5 (0.9/11.2)**	3.1 (−2.3/8.8)	**9.8 (2.1/18.1)**	3.1 (−2.8/9.3)	**8.1 (1.4/15.2)**
	0–4	**6.8 (1.9/12.1)**	4.0 (−1.8/10.2)	**12.3 (3.9/21.4)**	3.4 (−2.9/10.1)	**10.9 (3.6/18.7)**
	0–5	**6.9 (1.6/12.5)**	3.4 (2.8/10.0)	**14.1 (5.0/24.0)**	3.3 (−3.5/10.5)	**11.4 (3.6/18.9)**
	0–6	**6.0 (0.4/11.8)**	2.6 (−4.0/9.6)	**12.8 (3.2/23.3)**	3.2 (−4.0/10.9)	**9.3 (1.0/18.2)**
	0–7	**6.5 (0.5/12.8)**	3.4 (−3.6/11.0)	**12.53 (2.3/23.7)**	4.6 (−3.1/13.0)	8.7 (−0.1/18.1)

*Bold font indicates statistical significance (P <0.05)*.

**Figure 1:**
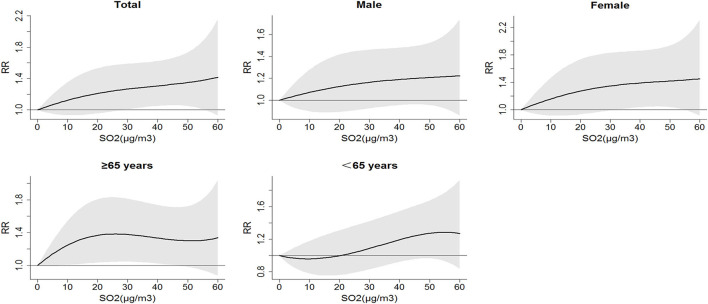
Relative risk (RR) of respiratory inpatient visits caused by different SO_2_ concentrations.

Figure 1 presents a positive exposure-response relationship between the SO_2_ concentration and the relative risk of respiratory inpatient visits. The exposure-response curves of the total population, men, women, and the elderly (≥65 years) were raised relatively faster in the range of 0–20 μg/m^3^, which meant that the risk of respiratory hospitalization increases rapidly under relatively low concentrations of SO_2_ exposure; the curves in the range of 20–50 μg/m^3^ were relatively flat, indicating that the risk of hospitalization remains at a relatively high level with the increase of SO_2_ concentration. The curve for younger people (<65 years) decreased slightly in the range of 0–10 μg/m^3^ and increased at higher concentration (20–50 μg/m^3^), which showed the risk of hospitalization for younger people's respiratory system does not change much under relatively low concentration of SO_2_ exposure.

**Figure 2:**
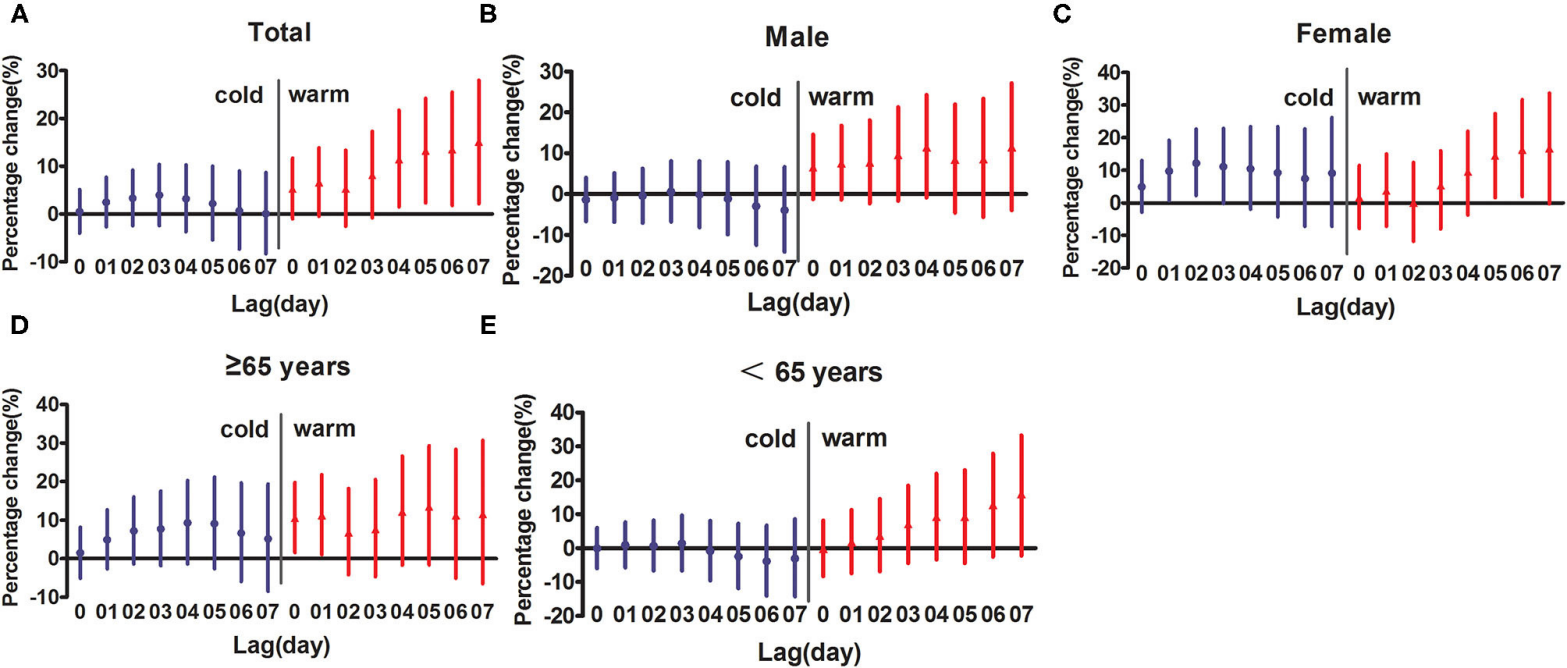
Percentage change (95% CI) of inpatient visits per 10 μg/m^3^ increase in concentrations of SO_2_ for different lag days in different seasons. **(A)** represents total people; **(B)** represents male; **(C)** represents female; **(D)** represents the elderly people (≥65 years); **(E)** represents younger people (<65 years).

As shown in Figure 2, the association between the SO_2_ concentration and the daily respiratory inpatient visits was stronger in the warm season (May–Oct) rather than in the cold season (Nov–Apr). For the elderly (≥65 years), the positive association between the SO_2_ concentration and the respiratory inpatient visits was statistically significant only in warm seasons. Table 4 summarizes the percent changes for daily respiratory inpatient visits associated with each 10 μg/m^3^ increase of SO_2_ concentration in multiple pollutant models. We used the data from lag3 and lag4 because the single-day lag effect of SO_2_ was statistically significant in the above lag days. The results from the multi-pollutant model indicated that the relationship between SO_2_ and respiratory inpatient visits was meaningless at lag3 after PM_10_ was controlled. When adjusted for all atmospheric pollutants, including PM_10_, NO_2_, O_3_, and CO, the association between SO_2_ and inpatient visits was still statistically significant at lag4. The effects of SO_2_ on respiratory inpatient visits were slightly decreased after adjusting for PM_10_, O_3_, and CO (2.6–3.3%) at lag4 and the percent change of inpatient visits became 3.8% after adjusting for NO_2_.

**Table 4:** Percentage change (95% CI) of inpatient visits associated with 10 μg/m^3^ increase of SO_2_ under multiple pollutant models.

**Lag day^a^**	**Model type**	**Percentage change**	**95% CI**
3	SO_2_	3.2	(0.6, 6.7)^*^
	SO_2_ + PM_10_	3.0	(−0.3, 7.2)
	SO_2_ + NO_2_	3.7	(0.7, 7.0)^*^
	SO_2_ + O_3_	2.9	(0.3, 5.9)^*^
	SO_2_ + CO	3.0	(0.8, 5.6)^*^
4	SO_2_	3.4	(0.4, 6.4)^*^
	SO_2_ + PM_10_	3.3	(0.3, 6.5)^*^
	SO_2_ + NO_2_	3.8	(1.1, 6.8)^*^
	SO_2_ + O_3_	2.6	(0.2, 5.6)^*^
	SO_2_ + CO	3.3	(1.1, 5.7)^*^

* *p <0.05*.

a *The association between SO_2_ exposure and daily respiratory inpatient visits was statistically significant at lag3 and lag4 in a single-lag model*.

## Discussion

Our research is a quantitative evaluation of the relevance between SO_2_ and daily respiratory inpatient visits in Ganzhou from 2017 to 2019 using the GAM and the DLNM. The results indicated that the elevation in SO_2_ concentration was significantly associated with an increase in respiratory inpatient visits, especially in the warm season (May–Oct), women, and elderly (≥65 years) subgroups. This association was stable after adjusting for other atmospheric pollutants. The exposure-response curves of the SO_2_ concentrations and the relative risk of respiratory inpatient visits were nearly non-linear with no obvious thresholds.

This study showed significant cumulative effects of SO_2_ concentration in a single pollutant model, with the peak at lag0–5. At this point, per 10 μg/m^3^ elevation of SO_2_ concentration was associated with a 6.9% (95% CI: 1.6–12.5%) increment in daily respiratory inpatient visits. The results of the previous studies were consistent with ours, indicating that the SO_2_ concentration was positively related to respiratory diseases (21). Early *in vivo* studies have observed that SO_2_ exposure causes bronchoconstriction and induces respiratory diseases (22). A study conducted in Thailand indicated that a 10 μg/m^3^ increase in the SO_2_ concentration was associated with a 1.83% (95% CI: 1.2–2.4%) enhancement in total respiratory hospital admissions at lag0–4 (23). Similarly, the positive relationship between respiratory admissions and SO_2_ was observed at lag 4 [relative risk (RR): 1.12; 95% CI: 1.05–1.21] in Malaysia (24). A systematic review also suggested that every 10 μg/m^3^ increase of the SO_2_ levels corresponded to 0.7% (95% CI: 0.1–1.4%) increment respiratory morbidity at lag0 (25). An ecological study reported that in Shenyang, a typical heavily polluted city in China, per 10 μg/m^3^ increase in SO_2_ was related to 0.7% (95% CI: 0–1.4%) increase in respiratory admissions at lag0 (26). The earliest observed positive relation between SO_2_ exposure and respiratory inpatient visits in our study was at lag3 in a single-day lag model. After comparing the average SO_2_ concentrations in Ganzhou (18.5 μg/m^3^) and Shenyang (55 μg/m^3^) during the study period, we speculated that the exposure of higher concentrations of SO_2_ had intraday effects on the respiratory health, while the exposure of relatively low concentrations of SO_2_ had a delayed effect.

In this study, the earliest observed association between the SO_2_ concentration and the daily inpatient visits with respiratory diseases was at lag0–3 in the multi-day lag model. Conversely, some earlier studies indicated that the strongest effects were observed on the current day (25) or lag0–1 (23). A plausible explanation for the longer-than-normal hysteresis effect observed in Ganzhou may be that long-term exposure to SO_2_ leads to a decrease in the sensitivity of residents to SO_2_. In addition, the differences in the estimated effects and lagged patterns of SO_2_ may be related to different outcome indicators of the study, local economic development, and the gender structure of the total population in different regions. A retrospective study from Switzerland suggested that the relationship between the SO_2_ exposure and the hospital admissions for respiratory diseases was not statistically significant (27). This might reflect the difference in population sensitivity caused by different cultural backgrounds and dietary structures.

The stratified analysis suggested that women and elderly (≥65 years) are more sensitive to SO_2_ exposure, which is consistent with previous studies (28, 29). An observational study conducted in Lanzhou, China, found that the estimated effect size of SO_2_ on respiratory hospital admissions was slightly larger in women than in men (30). Physiological factors, such as lower red blood cell count and higher airway response, may be important reasons that cause women to be more sensitive to atmospheric pollutant exposure (31, 32). Moreover, the health differences according to sex may be affected by smoking, drinking, and other unhealthy living habits and occupational environment. Interestingly, in an earlier multi-city time series analysis, gender differences in the impact of SO_2_ were not observed, and this study pointed out that the insignificant gender differences may be due to factors, such as study design, sample size, and modeling strategy (33).

Consistent results have been reported on the effects of ambient air pollution on specific age groups. Qiu et al. found that each 10 μg/m^3^ increase in SO_2_ corresponds to a 3.4% (95% CI: 2.3–4.5%) increase in overall respiratory hospital admissions among the elderly (>65 years) in the Sichuan Basin (33). Similarly, earlier studies observed that SO_2_ was more associated with hospital admissions for respiratory diseases in older adults (>65) than in other age subgroups (26). The elderly are more susceptible to pathogen exposure due to the weaker immune defenses and respiratory functions (34). This may be an important reason for their increased sensitivity to SO_2_ exposure.

The health effects of SO_2_ in different seasons have long been debated. The results showed a positive relation between SO_2_ and daily respiratory inpatient visits during the warm season. Two study indicated that the relationship between SO_2_ and respiratory disease morbidity could be observed only during the cold season (heating season) (30, 35). It was worth mentioning that the research site is located in northern China, where temperatures are well below freezing in winter, and fossil fuels need to be burned for heating. The SO_2_ level in the cold season in northeast China was much higher than that in the warm season. However, there was no statistical difference in the SO_2_ concentration in Ganzhou during the cold and warm seasons (Supplementary Table 1). A recent study, also from southern China, was consistent with our findings, confirming a statistically significant association between SO_2_ and respiratory disease mortality during warm seasons (36). People in warm seasons tend to spend longer times outdoors. Even indoors, residents often open windows during warm seasons, and the indoor SO_2_ level is closer to the ambient SO_2_ level. Moreover, the different meteorological conditions (especially extreme temperatures) in different regions may be one of the important reasons for the variability (37–39).

Understanding the exposure-response curves of atmospheric pollutants is very important for the making of environmental and public health policies. As shown in Figure 1, we found that even at very low exposure levels, a positive correlation between SO_2_ and daily respiratory inpatient visits could be observed in the elderly subgroups. Similarly, several studies demonstrated adverse effects of SO_2_ at relatively low concentrations (40, 41). The health effect of SO_2_ as a single pollutant or with other atmospheric pollutants has long been controversial. In this study, the association between the SO_2_ concentration and the respiratory inpatient visits was stable after adjusting for other air pollutants at lag4. A study in Japan by Yorifuji et al. (42) also suggested that the relationship between SO_2_ exposure and respiratory system mortality can still be observed after adjusting for atmospheric pollutants, such as nitrogen dioxide and particulate matter. Yang et al. (43) proposed that multi-pollutant models increase the standard error, so the effects of multi-pollutant models tend to be slightly lower than those of single-pollutant models, which was consistent with the results of this study. However, a systematic review suggests that after adjusting for PM_10_ and NO_2_, no association between respiratory morbidity and SO_2_ had been observed (25). It is worth noting that there is a strong correlation between atmospheric pollutants, and it is difficult to accurately assess the adverse effects of a single pollutant even if multi-pollutant models are used (44).

In this study, the generalized additive model was adopted to adjust for the influence of confounding factors, such as long-term trends of the time, meteorological conditions, weekend, and holiday effects. Our study revealed a positive correlation between the SO_2_ concentration and the morbidity of respiratory diseases in China. Nevertheless, the study has several limitations. Firstly, the average concentration at fixed air monitoring stations was used as a proxy for individual exposure, which may underestimate the adverse effects of SO_2_ (45). Secondly, even if multi-pollutant models were used, the independent effects of SO_2_ could not be fully explored because there are no data on other particulate pollutants except PM_10_ in this study. Thirdly, the main model of this study did not include several confounding factors, such as daily activities and socioeconomic status, which may not fully reflect the association between SO_2_ and respiratory inpatient visits.

The relationship between the elevated SO_2_ concentrations and the daily respiratory inpatient visits was observed in Ganzhou, a subtropical city in southern China, even though the average daily SO_2_ concentrations were lower than the minimum allowable exposure concentration set by the WHO (46) and China (47). We call on scholars in different regions to research on the health damage caused by common air pollutants to provide a theoretical basis for local health promotion and environmental health policy formulation. Future investigations should require more rigorous experimental designs (e.g., a combination of animal experiments and population epidemiological investigations) to identify the subgroups susceptible to respiratory damage caused by the SO_2_ exposure.

## Conclusion

Our study indicated that SO_2_ concentration is positively associated with daily inpatient visits for respiratory disease. The association is closer in women, elderly people (≥65 years), and warmer seasons (May–Oct) subgroups. These results provide further evidence to support the potential health effects of exposure to SO_2_. We hope that our study can remind researchers and managers to pay more attention to the adverse effects of SO_2_ in developing countries.

## Data Availability Statement

The data analyzed in this study is subject to the following licenses/restrictions: the disclosure and use of inpatient information requires the consent of the relevant departments of the hospital. Requests to access these datasets should be directed to https://www.gyyfy.com/.

## Author Contributions

XZho carried out literature retrieval, determined the research direction and data analysis according to the existing literature, and wrote the article. YG participated in the data collection of articles and assisted in statistical analysis. DW and WC explained the data results, discussed them in combination with existing articles, and assisted in writing the first draft of the article. XZha was mainly responsible for contacting the hospital to provide the data required for this study and putting forward modification opinions on the first draft of the article. All authors read and approved the final manuscript.

## Funding

This work was funded by Gannan Medical University (Fund No. QD201901) and Department of Education of Jiangxi Province (Fund No. GJJ190786).

## Conflict of Interest

The authors declare that the research was conducted in the absence of any commercial or financial relationships that could be construed as a potential conflict of interest.

## Publisher's Note

All claims expressed in this article are solely those of the authors and do not necessarily represent those of their affiliated organizations, or those of the publisher, the editors and the reviewers. Any product that may be evaluated in this article, or claim that may be made by its manufacturer, is not guaranteed or endorsed by the publisher.
