# Supplementary material for: Association Between Sulfur Dioxide and Daily Inpatient Visits With Respiratory Diseases in Ganzhou, China: A Time Series Study Based on Hospital Data
Source: Front Public Health. 2022 Mar 31;10:854922. doi: 10.3389/fpubh.2022.854922 (PMC9008542; doi:10.3389/fpubh.2022.854922)
Supplement: Supplementary file 1 [file Table_1.DOCX]

Supplementary table 1 : Statistical description of atmospheric pollutant concentrations in warm and cold seasons

|  | Season | min | P_25_ | median | P_75_ | max | Z-value | P-value |
| --- | --- | --- | --- | --- | --- | --- | --- | --- |
| PM_10_ | Cold | 11 | 39 | 66 | 99 | 258 | -7.807 | <0.001 |
|  | Warm | 14 | 37 | 50 | 64 | 122 |  |  |
| SO_2_ | Cold | 2 | 10 | 16 | 26 | 73 | -1.626 | 0.104 |
|  | Warm | 3 | 11 | 16 | 21 | 64 |  |  |
| NO_2_ | Cold | 8 | 19 | 27 | 37 | 84 | -16.258 | <0.001 |
|  | Warm | 8 | 13 | 16 | 22 | 56 |  |  |
| O_3_ | Cold | 5 | 35 | 55 | 80 | 158 | -12.012 | <0.001 |
|  | Warm | 17 | 61 | 80 | 102 | 194 |  |  |
| CO | Cold | 0.7 | 1.2 | 1.4 | 1.7 | 2.9 | -14.156 | <0.001 |
|  | Warm | 0.6 | 0.9 | 1.1 | 1.3 | 1.8 |  |  |
